# Blood Pressure Level Is Associated With Changes in Plasma Aβ_1 –40_ and Aβ_1–42_ Levels: A Cross-sectional Study Conducted in the Suburbs of Xi’an, China

**DOI:** 10.3389/fnagi.2021.650679

**Published:** 2021-06-04

**Authors:** Meilin She, Suhang Shang, Ningwei Hu, Chen Chen, Liangjun Dang, Ling Gao, Shan Wei, Kang Huo, Jingyi Wang, Jin Wang, Qiumin Qu

**Affiliations:** ^1^Department of Neurology, The First Affiliated Hospital of Xi’an Jiaotong University, Xi’an, China; ^2^Department of Neurology, Yulin Hospital of Traditional Chinese Medicine, Shaanxi, China; ^3^Huyi Hospital of Traditional Chinese Medicine, Xi’an, China

**Keywords:** Alzheimer’s disease, plasma β-amyloid level, blood pressure, apolipoprotein E, hypertension

## Abstract

**Objectives**: Amyloid-β (Aβ) deposition in the brain is the hallmark of Alzheimer’s disease (AD) pathology. Hypertension is a risk factor for AD, but the effects of hypertension on Aβ deposition are not fully determined. Considering peripheral Aβ closely relates to Aβ deposition in the brain, we investigated the relationships between blood pressure (BP) level and plasma Aβ concentrations.

**Methods**: One-thousand and sixty-nine participants (age above 45) from a village in the suburbs of Xi’an, China were enrolled. Questionnaires and validated Chinese versions of the Mini-Mental State Examination (MMSE) were used to collect information about vascular risk factors and assess cognition function. The apolipoprotein E (ApoE) genotype was detected using PCR and sequencing. Plasma Aβ levels were measured using ELISA. The associations between BP and plasma Aβ levels were analyzed by using multivariate linear regression.

**Results**: Plasma Aβ_1–40_ level was higher in high BP group than that in normal BP group (53.34 ± 8.50 pg/ml vs. 51.98 ± 8.96 pg/ml, *P* = 0.013), in high SBP group than that in normal SBP group (53.68 ± 8.69 pg/ml vs. 51.88 ± 8.80 pg/ml, *P* = 0.001) and in high MABP group than that in normal MABP group (54.05 ± 8.78 pg/ml vs. 52.04 ± 8.75 pg/ml, *P* = 0.001). After controlling for the confounding factors, SBP (*b* = 0.078, *P* < 0.001), DBP (*b* = 0.090, *P* = 0.008) and MABP (*b* = 0.104, *P* < 0.001) correlated with plasma Aβ_1–40_ level positively in ApoE ε4 non-carriers, but not ApoE ε4 carriers.

**Conclusions**: Elevated BP levels were associated with increased plasma Aβ_1–40_ levels in middle-aged and elderly ApoE ε4 non-carriers.

## Introduction

Alzheimer’s disease (AD) is the most common cause of dementia, affecting more than 33.9 million people worldwide (Barnes and Yaffe, 2011). The deposition of amyloid-β (Aβ) in the brain is the main pathological characteristic of AD (Karran et al., 2011), and the amyloid cascade hypothesis is widely considered to underlie the pathogenesis of AD (Karran et al., 2011). Studies have found that elevated blood pressure (BP) levels in midlife may be related to the development and progression of AD in later life (Kivipelto et al., 2001; Qiu et al., 2005; Gottesman et al., 2017; Walker et al., 2019). According to recent studies, hypertension is associated with an increased Aβ burden in the brain (Ingmar and Deborah, 2013). Based on accumulating evidence, elevated BP may impair the clearance of Aβ and increase Aβ production in both the peripheral circulation and the central nervous system (Faraco and Iadecola, 2013).

Plasma Aβ, the source of which is mainly brain efflux *via* low-density lipoprotein receptor-related protein-1 (LRP1) through the blood-brain barrier (BBB) or glymphatic system (Roberts et al., 2014), is closely related to brain Aβ deposition (Vergallo et al., 2019). A complex equilibrium is believed to exist between the amyloid burden in the brain and plasma Aβ levels in both animal models and healthy individuals (DeMattos et al., 2002b; Giedraitis et al., 2007). The continuous translocation of Aβ from the brain parenchyma to the peripheral blood is essential for preventing Aβ accumulation and reducing the Aβ burden in the brain (DeMattos et al., 2001; Matsuoka et al., 2003). Plasma Aβ levels were recently reported to be associated with the incidence of AD (Ertekin-Taner et al., 2008; Lambert et al., 2009; Pérez-Grijalba et al., 2019). However, the relationship between the blood pressure (BP) level and plasma Aβ level is currently unclear. In the present study, we investigated the relationships between the parameters of BP and plasma Aβ levels in middle-aged and older individuals in the general population.

## Materials and Methods

### Study Population

**Figure 1:**
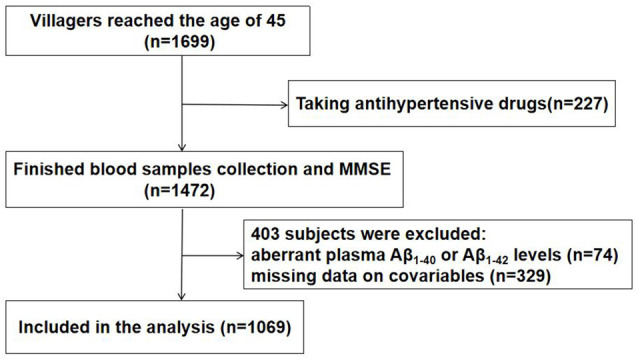
Flow chart of participant selection. Aβ, amyloid-β.

This is an ongoing population-based study designed to determine the potential vascular factors for AD in the general population. We used the cluster sampling method from October 2014 to March 2015 to make a face-to-face questionnaire survey on all the permanent residents in Qubao village, Huyi District, Xi’an City, and conducted a household survey on the disabled. The lifestyle and population composition of the village are similar to other rural areas in Xi’an. This cluster sampling method is consistent with statistical rules and has been proved to be reliable in our previous publications (Wang et al., 2018). The inclusion criteria were the following: (1) age 45 years or older, (2) registered permanent resident living in Qubao Village for more than 3 years, and (3) consented to participate in the study. The exclusion criteria were the following: (1) individuals who suffered from severe kidney disease, cancer, chronic liver conditions or a severe heart, pulmonary, or hematological disease, (2) individuals taking anti-hypertensive medicine, and (3) had missing covariates, or at least one aberrant plasma Aβ_1–40_ or Aβ_1–42_ level. The flow chart of study is shown in Figure 1.

Among the 1, 699 residents living in the village and older than 45 years, 227 were taking anti-hypertension medicine, 329 had missing covariates, and 74 had at least one aberrant plasma Aβ_1–40_ or Aβ_1–42_ level (exceeding ± 3 SDs from the mean). After all the exclusions, a final count of 1, 069 subjects was included in the study. The protocols used were reviewed and approved by the Ethics Committee of the First Affiliated Hospital of Xi’an Jiaotong University (No: XJTU1AF2014LSK-111).

### Data Collection

Subjects completed standardized questionnaires of general information to collect demographic data (age, sex, and education levels) and lifestyle habits (alcohol abuse, self-reported smoking history as current/former/never, and physical activity level) and underwent tests to determine the levels of multiple laboratory markers. We also recorded the medical history of cardiovascular disease, and transient ischaemic attack (TIA) or stroke. Additionally, we measured height, weight, BMI {which was calculated as [weight (kg)]/[height (m)^2^]}, and the pulse rate. The following vascular risk factors were measured: hypertension (defined as a mean systolic blood pressure measurement ≥140 mm Hg or diastolic blood pressure ≥ 90 mm Hg, a self-reported medical diagnosis, or use of antihypertensive drug therapy), diabetes (fasting serum glucose level ≥7.0 mmol/L, or use of diabetic medication or insulin.), and hyperlipidemia (fasting serum cholesterol concentration >5.18 mmol/L, serum triglyceride concentration >1.70 mmol/L, serum LDL cholesterol concentration >3.37 mmol/L, serum HDL cholesterol concentration <1.04 mmol/L, a self-reported medical diagnosis, or use of medication). Laboratory test parameters were measured in the clinical laboratory of The First Affiliated Hospital of Xi’an Jiaotong University.

### Cognitive Evaluation

The Mini-Mental State Examination (MMSE) was used to assess global cognition (Katzman et al., 1988) in a quiet room. Examiners underwent standardized training prior to the study, and consistency between the examiners was evaluated in a pilot study (kappa: 0.76–1). We chose an MMSE score lower than the cut-off value set by Katzman et al. (1988) as the criterion for cognitive impairment; specifically, the cut-off value was ≤17 for the uneducated, ≤20 for individuals with primary school education, and ≤24 for individuals educated at the junior high school level or above.

### BP Measurements

Blood pressure measurements were obtained during the inclusion interview for this study. Two brachial blood pressure measurements were recorded twice in a seated position after subjects had rested for at least 10 min. The instruments were a manual mercury sphygmomanometer with an appropriate-sized cuff (Shanghai Medical Instruments Co. Shanghai, China). Korotkoff phases 1 and 5 established the levels of systolic blood pressure (SBP) and diastolic blood pressure (DBP), respectively. The average of two measurements was used for analysis. The mean arterial pressure (MABP) was defined as [(SBP + DBP)/3].

Four variables, BP, SBP, DBP, and MABP, were used as indicators of the blood pressure level. A high BP was defined as a mean SBP ≥140 mm Hg or DBP ≥ 90 mm Hg. A high SBP was defined as a mean SBP ≥140 mm Hg. A high DBP was defined as a mean DBP ≥ 90 mm Hg. A high MABP was defined as a mean MABP ≥ 105 mm Hg.

### Detection of Plasma β-Amyloid Levels

Fasting blood samples (8:00–9:00 AM) were collected into vacutainers containing EDTA, an anticoagulant, centrifuged at 3000 g for 10 min, and stored at -80°C until use. Plasma Aβ_1–40_ and Aβ_1–42_ levels were measured with sandwich enzyme-linked immunosorbent assay kits (ELISA, Yuanye Co., Shanghai, China) as previously described (Wang et al., 2018). Measurements were performed by professionals in an independent laboratory of neurology. All measures were conducted under standardized conditions. The Aβ_1–42_ assay does not cross-react with Aβ_1–40_, and the Aβ_1–40_ assay does not cross-react with Aβ_1–42_. The recovery of the Aβ_1–42_ assay ranges from 75% to 106%, with an average value of 92%. The recovery of the Aβ_1–40_ assay ranges from 78% to 105%, with an average value of 90%. Measurements by recording the absorbance at 450 nm on a RT-6000 analyzer (Rayto Co., Shenzhen, China), and then concentrations were calculated using the standard curve. The limit of detection for each assay was 1.0 pg/ml. All samples were measured in duplicate and the results were averaged. The intra-assay and inter-assay coefficients of variation were less than 7% and 9%, respectively.

### Apolipoprotein E Genotyping

Polymerase chain reaction (PCR) was used to amplify the target gene fragment and the ApoE genotype was determined by one generation sequencing in 961 participants. ApoE genotype was defined based on the number of ε4 alleles. Based on the ApoE genotype, the participants were classified into the ApoE ε4 (−) group (E2/2, E2/3, and E3/3) and the ApoE ε4 (+) group (E2/4, E3/4, and E4/4).

### Data Analysis

All of the data were analyzed with SPSS 22.0 software. All graphs were drawn using GraphPad Prism software version 5.0. Quantitative variables are reported as the means ± SD or medians (interquartile ranges), and qualitative variables are reported as numbers (percentages). Unpaired Student’s *t*-tests were used to analyses data with an approximately normal distribution, the Mann-Whitney U-test was used to compare data with skewed distributions, and the c^2^ or Fisher’s exact test was used for categorical data. However, plasma triglyceride and fasting blood glucose (FBG) levels were log transformed prior to analysis, as they displayed skewed distributions. Then, unpaired Student’s *t*-tests were used to compare the differences in plasma Aβ levels in the subgroups stratified by BP parameters. Partial correlation coefficients and multivariate linear regression models were used to evaluate the associations between BP levels and Aβ levels after adjusting for the confounding factors. Model 1 was adjusted for age and sex, and model 2 was additionally adjusted for the BMI, pulse rate, ApoEε4 carrier status, log-transformed fasting blood glucose level, log-transformed triglyceride level, total cholesterol level, high-density lipoprotein level, smoking status, drinking status, physical activity level, stroke, transient ischaemic attack, and heart disease. The linear correlation and regression analyses were performed to explore the potential effect of ApoE genotype on the relationships. Potential confounders identified in previous studies that might affect BP and plasma Aβ levels were considered. A *P* value of <0.05 (two-tailed) was considered statistically significant.

## Results

### Characteristics of the Study Population

The characteristics of the study population are presented in Table 1. The high BP group was older and included a higher proportion of individuals with diabetes, dyslipidemia, and a lack of physical activity. This group also presented higher values for the BMI, pulse rate, fasting blood glucose level, and serum cholesterol and triglyceride concentrations. A significantly lower MMSE score was recorded by the high BP group, but the ApoE ε4 allele carrier status was not different between the high BP group and the normal BP group.

**Table 1:** Characteristics of the total study population and the population stratified by blood pressure.

Characteristic	Total sample (*n* = 1,069)	Normal BP group (*n* = 625)	High BP group (*n* = 444)	*P*-Value
Age, y (mean ± SD)	57.4 ± 9.2	55.8 ± 8.7	59.6 ± 9.3	<0.001
Female, %	622 (58.2%)	366 (58.6%)	256 (57.7%)	0.801
Educational level, y	6.1 ± 3.5	6.4 ± 3.3	5.7 ± 3.6	<0.001
Medical history, *n* (%)				
Diabetes mellitus	124 (11.6%)	59 (9.4%)	65 (14.6%)	0.009
Heart disease	56 (5.2%)	32 (5.1%)	24 (5.4%)	0.836
Stroke or TIA	79 (7.4%)	37 (5.9%)	42 (9.5%)	0.023
Dyslipidemia	545 (51.0%)	291 (46.6%)	254 (57.2%)	0.001
Blood pressure				
SBP (mmHg)	131.0 ± 17.1	120.0 ± 9.4	146.7 ± 12.8	<0.001
DBP (mmHg)	81.3 ± 9.5	76.1 ± 6.1	88.6 ± 8.6	<0.001
MABP (mmHg)	97.9 ± 11.2	90.7 ± 6.5	107.9 ± 8.1	<0.001
ApoE ε4 carrier, *n* (%)^a^	153 (14.3%)	93 (14.9%)	60 (13.5%)	0.450
Personal history, *n* (%)				
Smoking	344 (32.2%)	193 (30.9%)	138 (31.1%)	0.122
Drinking	160 (15.0%)	92 (14.7%)	68 (15.3%)	0.788
Lack of physical activity	191 (17.9%)	97 (15.5%)	94 (21.2%)	0.019
BMI, kg/m^2^ (mean ± SD)	25.0 ± 3.2	24.6 ± 2.9	25.6 ± 3.4	<0.001
Pulse rate, bpm (mean ± SD)	75.0 ± 7.9	74.2 ± 7.5	76.1 ± 8.4	<0.001
Fasting blood glucose, mmol/L	5.39 (5.06, 5.77)	5.32 (5.00, 5.67)	5.45 (5.15, 5.96)	0.059
TG, mmol/L	1.42 (1.04, 1.99)	1.32 (1.00, 1.81)	1.59 (1.14, 2.18)	<0.001
TC, mmol/L	5.05 ± 0.97	4.98 ± 0.97	5.14 ± 0.95	0.010
HDL-c, mmol/L	1.42 ± 0.31	1.43 ± 0.31	1.40 ± 0.30	0.084
MMSE score	25 (24, 28)	27 (24, 28)	26 (23, 28)	0.003
Aβ_1–40_, pg/ml	52.54 ± 8.80	51.98 ± 8.96	53.34 ± 8.50	0.013
Aβ_1–42_, pg/ml	41.05 ± 6.64	41.12 ± 6.57	40.96 ± 6.73	0.707
Aβ_1–42_/Aβ_1–40_	0.80 ± 0.19	0.82 ± 0.19	0.79 ± 0.19	0.026

*^a^Data available for 961 participants. Numbers are mean (SE) or count (%). P values obtained from *t* test or *χ*^2^ test or ANOVA in comparision of high BP group and normal BP group. Abbreviations: TIA, transient ischemic attack; BMI, body mass index; TG, triglyceride; TC, total cholesterol; HDL-c, high-density lipoprotein cholesterol*.

### Comparison of Plasma Aβ Levels Between the High BP Group and Normal BP Group

**Figure 2:**
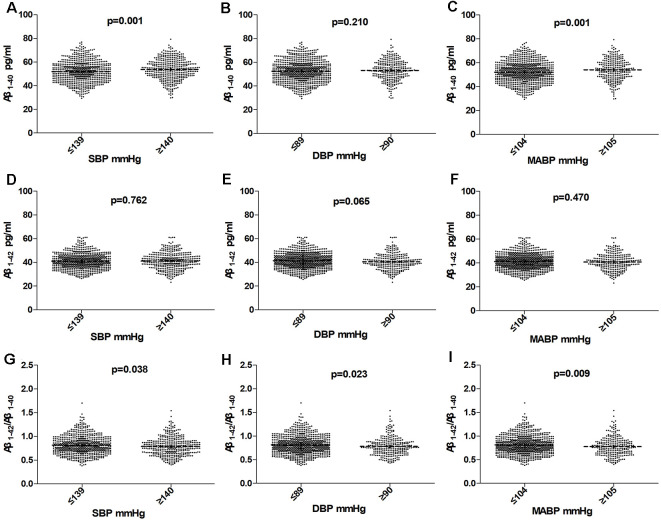
Comparision of Aβ_1–40_, Aβ_1–42_, and Aβ_1–42_/Aβ_1–40_ in subjects according to SBP **(A,D,G)**, DBP **(B,E,H)**, MABP **(C,F,I)** in the total population. Horizontal lines represent the mean and 95% CI. Abbreviations: BP, blood pressure; SBP, systolic blood pressure; DBP, diastolic blood pressure; MABP, mean arterial blood pressure.

As shown in Table 1 and Figure 2; plasma Aβ_1–40_ level was higher in the high BP group than that in the normal BP group (53.34 ± 8.50 pg/ml vs. 51.98 ± 8.96 pg/ml, *P* = 0.013), while the Aβ_1–42_/Aβ_1–40_ ratio was significantly lower (0.79 ± 0.19 vs. 0.82 ± 0.19, *P* = 0.026), but the Aβ_1–42_ level had no significant difference between the high BP group and normal BP group (*P* = 0.707). Furthermore, plasma Aβ_1–40_ level was higher in the high SBP group (53.68 ± 8.69 pmol/L vs. 51.88 ± 8.80 pmol/L, *P* = 0.001) and high MABP group (54.05 ± 8.78 pmol/L vs. 52.04 ± 8.75 pmol/L, *P* = 0.001), but Aβ_1–42_/Aβ_1–40_ ratio was lower in the high SBP (0.79 ± 0.19 vs. 0.81 ± 0.19, *P* = 0.038), high DBP (0.78 ± 0.19 vs. 0.81 ± 0.19, *P* = 0.023), and high MABP (0.78 ± 0.20 vs. 0.81 ± 0.19, *P* = 0.009) groups.

### The Association Between BP and Plasma Aβ Levels in the Multivariate Analysis

**Figure 3:**
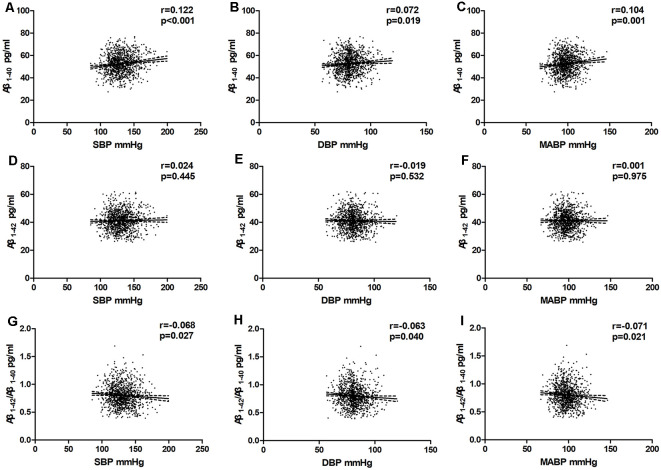
Partial linear correlations between plasma Aβ and SBP, DBP, MABP in total participants. Partial linear correlations of blood pressure (BP) with plasma Aβ_1–40_ levels **(A–C)** and plasma Aβ_1–42_ levels **(D–F)** and Aβ_1–42_/Aβ_1–40_ **(G–I)** are shown in the figure. Partial correlation coefficients and P values were obtained after adjustment for age, sex, BMI, pulse rate, log-transformed fasting blood glucose, log-transformed TG, TC, HDL-C, smoking, drinking, physical activity level, stroke, transient ischaemic attack (TIA), and heart disease.

In the total population, the relationship between BP and plasma Aβ levels was estimated using partial correlation analyses and multivariate regression analyses. As shown in Figure 3 and Table 2; plasma Aβ_1–40_ levels correlated with SBP (*r* = 0.106, β = 0.056, *P* = 0.001) and MABP (*r* = 0.082, β = 0.065, *P* = 0.008) positively after controlling for age and sex. After additionally controlling for all the confounding factors listed in Table 2, plasma Aβ_1–40_ levels were positively correlated with SBP (*r* = 0.122, β = 0.068, *P* < 0.001), DBP (*r* = 0.072, β = 0.071, *P* = 0.019) and MABP (*r* = 0.104, β=0.087, *P* = 0.001). The Aβ_1–42_/Aβ_1–40_ ratio was negatively correlated with SBP (*r* = −0.068, β = −0.001, *P* = 0.027), DBP (*r* = −0.063, β = −0.001, *P* = 0.040), and MABP (*r* = −0.071, β = −0.001, *P* = 0.021).

**Table 2:** Multiple linear regression models analysis of blood pressure components and plasma Aβ_1–40_, Aβ_1–42_, and Aβ_1–42_/Aβ_1–40_ ratio in total study subjects.

	Aβ_1–40_		Aβ_1–42_		Aβ_1–42_/Aβ_1–40_	
	β	*p*	β	*p*	β	*p*
Model 1						
SBP	0.056	0.001	0.008	0.507	−0.001	0.063
DBP	0.052	0.104	−0.017	0.418	−0.001	0.103
MABP	0.065	0.008	−0.002	0.894	−0.001	0.062
Model 2						
SBP	0.068	<0.001	0.010	0.445	−0.001	0.027
DBP	0.071	0.019	−0.014	0.532	−0.001	0.040
MABP	0.087	0.001	0.001	0.975	−0.001	0.021

*β, the unstandardized regression coefficient. Model 1: adjusted for age and sex. Model 2: adjusted for age, sex, BMI, pulse rate, log-transformed fasting blood glucose, log-transformed TG, TC, HDL-C, smoking, drinking, physical activity level, TIA, and heart disease*.

### The Effects of the ApoE ε4 Allele on Plasma Aβ Levels

As ApoE ε4 is the strongest genetic risk factor for AD, we investigated the effects of the ApoE ε4 allele on plasma Aβ levels. In the univariate analysis, ApoE ε4 non-carriers had a lower proportion of females than ApoE ε4 carriers, however other covariates (age, gender, MMSE score, education level, smoking status, drinking status, intensity of physical activity, hypertension, diabetes mellitus, coronary heart disease, MABP, and BMI) had no significant difference between the two groups. The plasma Aβ_1–40_ and Aβ_1–42_ concentrations and the Aβ_1–42_/Aβ_1–40_ ratio had no significant difference between ApoE ε4 non-carriers and ApoE ε4 carriers (Table 3).

**Table 3:** Characteristics of the subpopulation stratified by ApoE ε4 carrier status.

Characteristic	**Subpopulation** (*n* = 961^a^)	ApoEε4 non-carriers (*n* = 808)	ApoEε4 carriers (*n* = 153)	*P*-Value
Age, y (mean ± SD)	57.6 ± 9.2	57.5 ± 9.2	58.1 ± 9.2	0.509
Female, %	556 (57.9%)	453 (56.1%)	103 (67.3%)	0.010
Educational level, y	6.1 ± 3.5	6.1 ± 3.4	5.9 ± 3.6	0.435
Medical history, *n* (%)				
Diabetes mellitus	114 (11.9%)	95 (11.8%)	19 (12.4%)	0.817
Heart disease	50 (5.2%)	40 (5.0%)	10 (6.5%)	0.418
Stroke or TIA	74 (7.7%)	62 (1.7%)	12 (7.8%)	0.461
Dyslipidemia	489 (50.9%)	403 (49.9%)	86 (56.2%)	0.151
Blood pressure, mmHg				
SBP	131.2 ± 17.2	131.4 ± 17.2	130.3 ± 17.3	0.477
DBP	81.3 ± 9.6	81.5 ± 9.6	80.2 ± 9.4	0.120
MABP	97.9 ± 11.2	98.1 ± 11.2	96.9 ± 11.3	0.213
Personal history, *n*(%)				
Smoking	297 (30.9%)	259 (32.1%)	38 (24.8%)	0.132
Drinking	146 (15.2%)	130 (16.1%)	16 (10.5%)	0.075
Lack of physical activity	176 (18.3%)	148 (18.3%)	28 (13.3%)	0.996
BMI, kg/m2 (mean ± SD)	25.0 ± 3.2	25.0 ± 3.1	25.3 ± 3.5	0.338
Pulse rate, bpm(mean ± SD)	74.9 ± 7.9	74.8 ± 8.0	75.4 ± 7.7	0.417
Fasting blood glucose, mmol/l	5.40 (5.06, 5.76)	5.40 (5.06, 5.77)	5.71 (5.07, 5.78)	0.582
TG, mmol/l	1.44 (1.04, 2.01)	1.43 (1.04, 2.01)	1.50 (1.06, 2.09)	0.577
TC, mmol/l	5.04 ± 0.96	5.03 ± 0.97	5.13 ± 0.94	0.217
HDL-c, mmol/l	1.42 ± 0.31	1.42 ± 0.30	1.40 ± 0.32	0.501
Aβ_1–40_, pg/ml	52.45 ± 8.94	52.36 ± 8.90	52.93 ± 9.20	0.468
Aβ_1–42_, pg/ml	41.16 ± 6.70	41.01 ± 6.68	41.94 ± 6.78	0.116
Aβ_1–42_/Aβ_1–40_	0.81 ± 0.19	0.81 ± 0.19	0.81 ± 0.19	0.602

*Numbers are mean (SE) or count (%). *P* values obtained from *t* test or *χ*^2^ test or ANOVA in comparison of ApoE ɛ4 non-carriers and ApoE ɛ4 carriers. ^a^Data available for 961 participants, 961 participants agreed to perform gene testing. Abbreviations: TIA, transient ischemic attack; BMI, body mass index; TG, triglyceride; TC, total cholesterol; HDL-c, high-density lipoprotein cholesterol*.

### Multivariate Analysis of the Relationship Between BP and Plasma Aβ Levels in Individuals Stratified According to the ApoE ε4 Status

The multivariate linear regression analyses and partial correlation analyses were performed in subgroups stratified according to ApoE ε4 status. In ApoE ε4 non-carriers, after controlling for confounders, plasma Aβ_1–40_ levels correlated with SBP, DBP and MABP positively (*r*_SBP_ = 0.143, β_SBP_ = 0.078, *P* < 0.001; *r*_DBP_ = 0.093, β_DBP_ = 0.090, *P* = 0.008; and *r*_MABP_ = 0.126 β_MABP_ = 0.104, *P* < 0.001, respectively); the Aβ_1–42_/Aβ_1–40_ ratio correlated with SBP, DBP, and MABP negatively (*r*_SBP_ = −0.072, β_SBP_ = −0.001, *P* = 0.043; *r*_DBP_ = −0.066, β_DBP_ = −0.001, *P* = 0.063; and *r*_MABP_ = −0.074, β_MABP_ = −0.001, *P* = 0.037, respectively). However, among ApoE ε4 carriers, these relationships disappeared. These suggested that the association between BP and plasma Aβ levels may depend on the ApoE ε4 status (Table 4).

**Table 4:** Partial linear correlation analysis and multiple linear regression models of blood pressure components and plasma Aβ_1–40_, Aβ_1–42_, and Aβ_1–42_/Aβ_1–40_ ratio in subjects stratified by ApoE ε4 status.

	ApoE ε4 non-carriers (*n* = 808)									ApoE ε4 carriers (*n* = 153)								
		Aβ_1–40_			Aβ_1–42_			Aβ_1–42_/Aβ_1–40_			Aβ_1–40_			Aβ_1–42_			Aβ_1–42_/Aβ_1–40_		
	*r*	β	*p*	*r*	β	*p*	*r*	β	*p*	*r*	β	*p*	*r*	β	*p*	*r*	β	*p*
SBP	0.132	0.070	<0.001	0.034	0.014	0.335	−0.066	−0.001	0.062	−0.025	−0.014	0.762	−0.006	−0.002	0.944	0.018	<0.00	0.831
DBP	0.078	0.073	0.026	−0.008	−0.005	0.829	−0.059	−0.001	0.096	−0.107	−0.105	0.191	−0.022	−0.016	0.785	0.070	0.001	0.390
MABP	0.111	0.088	0.002	0.013	0.008	0.721	−0.067	−0.001	0.059	−0.073	−0.060	0.374	−0.016	−0.009	0.850	0.049	0.001	0.554
SBP	0.143	0.078	<0.001	0.039	0.016	0.266	−0.072	−0.001	0.043	−0.019	−0.011	0.823	−0.039	−0.017	0.650	−0.003	−4.153	0.968
DBP	0.093	0.090	0.008	<0.001	<0.001	0.991	−0.066	−0.001	0.063	−0.095	−0.098	0.262	−0.041	−0.030	0.629	0.052	0.001	0.542
MABP	0.126	0.104	<0.001	0.020	0.012	0.579	−0.074	−0.001	0.037	−0.064	−0.057	0.451	−0.043	−0.027	0.616	0.028	0.001	0.741

*r, the partial correlation coefficient. β, the unstandardized regression coefficient. Model 1: adjusted for age and sex. Model 2: adjusted for age, sex, BMI, pulse rate, log-transformed fasting blood glucose, log-transformed triglyceride, total cholesterol, high-density lipoprotein, smoking, drinking, physical activity level, stroke or transient ischemic attack, and heart disease*.

## Discussion

In this cross-sectional study, we investigated the relationships between multiple BP components and plasma Aβ levels and found that elevated BP levels were associated with increased plasma Aβ_1–40_ levels and decreased Aβ_1–42_/Aβ_1–40_ ratio in middle-aged and older villagers, even after controlling for other confounding factors. However, the association was only observed in ApoE ε4 non-carriers, but not ApoE ε4 carriers.

Several publications have suggested an association between plasma Aβ and BP levels (Fujiwaraa et al., 2003; Abdullah et al., 2009; Lambert et al., 2012; Ruiz et al., 2013; Wang et al., 2018). However, a consistent conclusion has not been drawn. A positive correlation between SBP and plasma Aβ_1–40_ levels (Abdullah et al., 2009; Lambert et al., 2012) or a negative correlation between SBP and plasma Aβ_1–40_ levels (Abdullah et al., 2009), as well as a positive correlation between DBP and plasma Aβ_1–42_ levels (Fujiwaraa et al., 2003) have been reported. These inconsistencies are likely due to the use of different inclusion criteria, exclusion criteria, and test methods. In those cross-sectional studies, investigators either used a case-control study design with a small sample size (Lambert et al., 2012; Ruiz et al., 2013) or only explored plasma Aβ_1–42_ and BP levels (Fujiwaraa et al., 2003).

Unlike the previous studies, our present study used a random cluster sampling method with a large sample size consisting of middle-aged and elderly individuals in the general population. All enrolled residents lived in the selected village for over 3 years with permanent residency. Plasma Aβ levels were detected using ELISA, which has been demonstrated as an accurate and dependable method (Katzman et al., 1988). We did a multiple analysis to adjusted for almost all identified potential confounder factors, including the ApoE genotype (Rodrigue et al., 2013; Giau et al., 2015), BMI (Qiu et al., 2005), MMSE score, and serum TC, TG and HDL-c levels (Matsuzaki et al., 2011). The relationships between plasma Aβ level and BP levels did not change. These results were similar to the three-city study by Lambert et al. (2012) which showed that elevated BP levels were associated with decreased plasma Aβ_1–42_/Aβ_1–40_ ratio.

The mechanism underlying the association between plasma Aβ and BP levels is not well understood. One possible mechanism is that BP may affect the deposition of Aβ in the brain. It has been reported that hypertension is associated with Aβ deposition in the brain in individuals with normal cognition. Animal experiments also observed a direct effect of hypertension on the deposition of Aβ in the brain (Cifuentes et al., 2015; Faraco et al., 2016). In PET imaging studies of middle-aged to old adults with normal cognition, Langbaum et al. revealed an association between elevated SBP and fibrillary Aβ levels in the brain (Langbaum et al., 2012). In addition, Rodrigue et al. (2013) observed a significant correlation between controlled hypertension and lower brain Aβ burden compared with un-medicated hypertension (Rodrigue et al., 2013). Aβ is generated primarily in the brain and cleared mainly through the blood-brain barrier from the central to the peripheral. Aβ levels are in dynamic equilibrium between CSF and plasma (DeMattos et al., 2002b). An increased efflux from the brain to plasma may result in higher plasma Aβ levels (DeMattos et al., 2002a). Thus, an elevated BP associated with higher plasma Aβ levels might relate to increased Aβ generation in the brains of individuals with normal cognition. And our previous studies showed that plasma Aβ_40_ levels positively correlated with sRAGE and sLRP1 (Gao et al., 2018), while BP positively correlated with Aβ_40_ and negatively correlated with sRAGE (Jiang et al., 2017). Therefore we speculate that BP changes the balance between peripheral and central Aβ affecting the levels of plasma Aβ_40_ and peripheral transporters. Further, it might have affected the peripheral clearance pathway of Aβ, increasing the deposition of Aβ in the brain and participating in the development of Aβ pathological process. These conjectures have not been confirmed and should be verified in experiments performed in appropriate animal models.

Another possible mechanism is that elevated BP may have affected the integrity of the BBB, increasing the Aβ transport. Research shows that hypertension damages the integrity of vascular endothelial cells and affects the vascular wall (Faraco and Iadecola, 2013). It has been hypothesized (Shah et al., 2012) that the vasoactivity of Aβ in combination with hypertension could hardly destroy the integrity of the vascular wall and reduce the clearance of Aβ in the brain which would lead to an inflammatory response and cell death. Despite all of this, the authors suggested that vascular integrity was an important part of the early trajectory. Importantly, blood pressure and plasma Aβ levels were measured 10–20 years before the diagnosis of AD, which indicates that early intervention on elevated BP might be very important for reducing AD caused by hypertension. This conclusion is also suggested by our results. Moreover, the elevated BP might impair the vascular clearance of Aβ and increase its cleavage from APP in both peripheral and the central nervous system to further facilitate the onset of AD (Ingmar and Deborah, 2013).

We did not find an association between BP and plasma Aβ_1–42_ levels, which might be due to the different physiological roles of Aβ_1–40_ and Aβ_1–42_. Aβ_1–42_ is insoluble and prone to fibrillate and deposit in senile plaques with greater neurotoxicity (Verbeek et al., 1997). While, Aβ_1–40_ is soluble, and has direct physiological or toxic effects on the blood vessel wall (Niwa et al., 2000). The levels of Aβ_1–40_, but not Aβ_1–42_, are markedly increased in patients with ischemic stroke (Lee et al., 2005) and diffuse SVD (Gomis et al., 2009). These indicated that Aβ_1–40_ is more closely relate to vascular disease than Aβ_1–42_.

In the subgroup analysis stratified according to the ApoE ε4 status, the association between plasma Aβ and BP levels were only observed in ApoE ε4 non-carriers, but not in ApoE ε4 carriers. The underlying mechanism is not clear. As the strongest genetic risk factor for AD, the ApoE ε4 allele was closely associated with the decrease of cerebral spinal flow Aβ and the increase of aggregation and deposition of cerebral Aβ in the brain (Liu et al., 2013; Giau et al., 2015). ApoE ε4 might compromise the effects of BP on plasma Aβ levels. Recent articles reported that the association between plasma Aβ levels and Aβ deposition in the brain was exactly observed in ApoE ε4 non-carriers (Swaminathan et al., 2014; Tateno et al., 2017). Additionally, Katsuya et al. reported that the prevalence of hypertension is lower in ApoE ε4 carriers (Katsuya et al., 2002). These indicated that the relationship between BP and plasma Aβ levels is dependent on ApoE ε4 states.

In this study, we measured plasma Aβ levels using an ELISA kit marketed by Yuanye Co (China). Compared to Aβ levels mentioned in other publications, the Aβ_42_ levels we determined are higher. We speculate this discrepancy might be due to the heterogeneity of the different populations studied and the measurement methods used and standards of Aβ that have not been validated to each other. Compared to the INNO-BIA assay, the ELISA Aβ_40_ levels measures are slightly lower, while the Aβ_42_ levels are slightly higher (Barnes and Yaffe, 2011). Our previous studies using the same ELISA kit produced credible results (Jiang et al., 2017; Gao et al., 2018; Wang et al., 2018); however, we acknowledge more rigorous data are needed to validate the comparisons.

## Conclusion

In summary, in this population-based cross-sectional study, we found that elevated BP levels were associated with increased plasma Aβ_1–40_ levels and decreased Aβ_1–42_/Aβ_1–40_ ratio in middle-aged and elderly villagers, particularly in ApoE ε4 non-carriers. Considering peripheral Aβ closely related to the deposition of Aβ in the brain, these results indicated that hypertension contribution to AD may be associated with peripheral Aβ transport dysfunction. The potential mechanism requires further validation.

## Limitations

First, in this cross-sectional study, BP and plasma Aβ levels were measured only once at a single time point, which is susceptible to physiological bias. Second, the cross-sectional design makes it difficult to determine causal relationships between plasma Aβ and BP levels. It is thus essential to conduct prospective cohort studies to identify the effects of BP on plasma Aβ levels. Third, we did not measure the levels of Aβ in the CSF and the deposition of Aβ in the brain simultaneously. Therefore, the increase in plasma Aβ levels may not indicate an increase of Aβ burden in the brain.

## Data Availability Statement

The raw data supporting the conclusions of this article will be made available by the authors, without undue reservation.

## Ethics Statement

The studies involving human participants were reviewed and approved by the Ethics Committee of the First Affiliated Hospital of Xi’an Jiaotong University (No: XJTU1AF2014LSK-111). The patients/participants provided their written informed consent to participate in this study.

## Author Contributions

MS and SS conceived the study, participated in its design, and wrote the manuscript. NH, CC, LD, LG, SW, JinW, KH, and JingW analyzed the data. QQ designed the study and revised the manuscript. All authors contributed to the article and approved the submitted version.

## Conflict of Interest

The authors declare that the research was conducted in the absence of any commercial or financial relationships that could be construed as a potential conflict of interest.

## Abbreviations

BP: blood pressure
SBP: systolic blood pressure
DBP: diastolic blood pressure
MABP: mean arterial blood pressure.
